# Draft Genome Sequences of Two Staphylococcus warneri Clinical Isolates, Strains SMA0023-04 (UGA3) and SMA0670-05 (UGA28), from Siaya County Referral Hospital, Siaya, Kenya

**DOI:** 10.1128/MRA.01595-18

**Published:** 2019-04-11

**Authors:** Gary Xie, Qiuying Cheng, Hajnalka Daligault, Karen Davenport, Cheryl Gleasner, Lindsey Jacobs, Jessica Kubicek-Sutherland, Tessa LeCuyer, Vincent Otieno, Evans Raballah, Norman Doggett, Harshini Mukundan, Douglas J. Perkins, Benjamin McMahon

**Affiliations:** a Biosecurity and Public Health, Bioscience Division, Los Alamos National Laboratory, Los Alamos, New Mexico, USA; b Center for Global Health, Department of Internal Medicine, University of New Mexico Health Sciences Center, Albuquerque, New Mexico, USA; c Theoretical Biology and Biophysics, Theoretical Division, Los Alamos National Laboratory, Los Alamos, New Mexico, USA; d Physical Chemistry and Applied Spectroscopy, Chemistry Division, Los Alamos National Laboratory, Los Alamos, New Mexico, USA; e University of New Mexico Laboratories of Parasitic and Viral Diseases, Kisumu, Kenya; f Department of Medical Laboratory Sciences, School of Public Health, Biomedical Sciences and Technology, Masinde Muliro University of Science and Technology, Kakamega, Kenya; Loyola University Chicago

## Abstract

We report the complete draft genome sequences of two Staphylococcus warneri clinical isolates, strains SMA0023-04 (UGA3) and SMA0670-05 (UGA28), each of which contains one chromosome and at least one plasmid. Isolate SMA0023-04 (UGA3) contains tetracycline efflux major facilitator superfamily (MFS) transporter (*tetK*), macrolide resistance (*msrC* and *mphC*), and beta-lactamase (*blaZ*) genes on its plasmids.

## ANNOUNCEMENT

Staphylococcus warneri, a Gram-positive human skin commensal bacterium, is catalase positive, oxidase negative, and coagulase negative. Like other coagulase-negative staphylococci, *S. warneri* rarely causes disease but may occasionally cause infection in immunocompromised patients (1). In this genome announcement, we report the draft genomes of two *S. warneri* strains, SMA0023-04 (UGA3) and SMA0670-05 (UGA28), isolated from the venous blood of pediatric patients at the Siaya County Referral Hospital (western Kenya) in 2004 and 2005, respectively. Isolate SMA0023-04 was from a febrile male pediatric patient 9.8 months of age who was HIV negative with Plasmodium falciparum malaria. Isolate SMA0670-05 was from a febrile female patient 7.93 months of age who was HIV negative with Plasmodium falciparum malaria.

Prior to any treatment interventions, blood was collected upon admission into a pediatric Isolator 1.5 microbial tube (Wampole Laboratories, Cranbury, NJ, USA) and cultivated at 35°C for 18 to 24 hours in 5% CO_2_ on 5% sheep blood agar. Bacterial DNA was extracted from a pure culture using the UltraClean microbial DNA isolation kit (Qiagen, Germantown, MD, USA) according to the manufacturer's instructions, with minimal modifications. The library was prepared from 100 ng of bacterial DNA by using an NEBNext Ultra DNA library prep kit for an Illumina instrument (New England Biolabs, Ipswich, MA, USA). *S. warneri* SMA0023-04 (UGA3) and SMA0670-05 (UGA28) were draft sequenced using a MiSeq version 2 500-cycle sequencing kit (Illumina, San Diego, CA, USA), generating 22,598,550 and 4,245,154 paired-end 251-bp reads resulting in 738- and 179-fold coverage, respectively. In addition, SMA0023-04 and SMA0670-05 contain at least one rep7-type and one rep20-type plasmid, respectively, findings which were supported by high coverage of *S. warneri* SG1 plasmids using BWA version 0.7.2 (2) read mapping from SMA0023-04 and SMA0670-05 as follows: 97.04% and 97.01% of *S. warneri* SG1 plasmid pvSw3, 58.79% and 59.98% of SG1 plasmid pvSw2, 56.17% and 67.57% of pvSw5, 38.84% and 26.79% of pvSw4, 37.66% and 26.17% of pvSw1, 25.40% and 43.69% of pSZ4, and 15.53% and 15.32% of pvSw6, respectively. Data quality was assessed and the data files were filtered and trimmed with FaQCs version 1.3 (3) and then assembled with Velvet version 1.2.08 (4, 5) and IDBA version 1.1.0 (6). The consensus sequences were computationally shredded and reassembled with Phrap version SPS-4.24 (7, 8) to allow some manual editing with Consed (9), resulting in 25 and 67 final contigs of >200 bp (99.50% and 95.89% of the reads), with *N*_50_ values of 480,873 bp and 665,039 bp for SMA0023-04 and SMA0670-05, respectively. These contigs cover 89.26% and 91.77% of the *S. warneri* SG1 chromosome (GenBank accession number NC_020164), respectively (10), using Mummer alignment version 3.0 (11). The draft genomes of SMA0023-04 and SMA0670-05 consist of 2,466,813- and 2,555,257-bp sequences, with average G+C contents of 32.5% and 32.6%, respectively. Annotations were completed at LANL with an automated system using the Ergatis workflow manager version 2.0 (12) and in-house scripts.

In addition to 60 tRNA genes and 7 rRNA genes in the genome of each isolate, there are 2,492 and 2,552 predicted protein coding genes within the genomes of SMA0023-04 (UGA3) and SMA0670-05 (UGA28), respectively. Of these, 40% and 39% of the protein-coding genes were annotated in a SEED subsystem (13), whereas 60% and 61% were not annotated in a SEED subsystem, respectively; 682 and 782 genes were annotated as hypothetical proteins in SMA0023-04 and SMA0670-05, respectively. Of all the predicted genes, 2,247 are in common among SMA0023-04, SMA0670-05, and the *S. warneri* SG1 chromosome genomes, with 109, 168, and 111 genes being unique to SMA0023-04, SMA0670-05, and *S. warneri* SG1, respectively. Twenty-three, 22, and 17 genes are associated with resistance to antibiotics in SMA0023-04, SMA0670-05, and *S. warneri* SG1, respectively. Unlike other methicillin-susceptible coagulase-negative staphylococcus (CoNS) strains that are highly sensitive to various antimicrobial drugs (14, 15), SMA0023-04 has displayed resistance to tetracycline, macrolide, and ampicillin using the disk diffusion method (16). These observations are consistent with the presence of the tetracycline efflux major facilitator superfamily (MFS) transporter, PC1 beta-lactamase, ABC-efflux pump, and macrolide phosphotransferase encoded by *tetK*, *blaZ*, *msrC*, and *mphC* genes, respectively, on the SMA0023-04 (UGA3) plasmid.

### Data availability.

The GenBank accession numbers for Staphylococcus warneri SMA0023-04 (UGA3) and SMA0670-05 (UGA28) are NWUB00000000 and NWUA00000000, respectively. This BioProject (PRJNA407975) has been assigned BioSample numbers SAMN07671984 and SAMN07671985 and SRA accession numbers SRR8655129 and SRR8655130 for UGA3 and UGA28, respectively.
